# HiCBricks: building blocks for efficient handling of large Hi-C datasets

**DOI:** 10.1093/bioinformatics/btz808

**Published:** 2019-11-07

**Authors:** Koustav Pal, Ilario Tagliaferri, Carmen Maria Livi, Francesco Ferrari

**Affiliations:** 1 IFOM, The FIRC Institute of Molecular Oncology, Milan, Italy; 2 Institute of Molecular Genetics, National Research Council, Pavia, Italy

## Abstract

**Summary:**

Genome-wide chromosome conformation capture based on high-throughput sequencing (Hi-C) has been widely adopted to study chromatin architecture by generating datasets of ever-increasing complexity and size. HiCBricks offers user-friendly and efficient solutions for handling large high-resolution Hi-C datasets. The package provides an R/Bioconductor framework with the bricks to build more complex data analysis pipelines and algorithms. HiCBricks already incorporates functions for calling domain boundaries and functions for high-quality data visualization.

**Availability and implementation:**

http://bioconductor.org/packages/devel/bioc/html/HiCBricks.html.

**Supplementary information:**

Supplementary data are available at *Bioinformatics* online.

## 1 Introduction

High-throughput sequencing (Hi-C) allows probing physical proximity between potentially any pair of genomic loci and has been widely adopted to characterize chromatin structure and function (Lieberman-Aiden *et al.*, 2009). Several Hi-C protocol variations and data analysis methods have been proposed (Forcato *et al.*, 2017). Moreover, a rapid escalation in the size and complexity of datasets has been causing challenges in data analysis (Pal *et al.*, 2019), data handling and interoperability (Marti-Renom *et al.*, 2018).

Large high-resolution Hi-C datasets, such as mammalian genomes binned at Kb resolution (Bonev *et al.*, 2017; Rao *et al.*, 2014), pose-specific challenges to computational biologists. First, there’s a lack of a standard data format for Hi-C contact matrices. The solutions adopted in literature and bioinformatic tools include text files with 2D matrices, sparse matrices represented in other tabular formats, as well as tool-specific binary formats such as ‘.mcool’ or ‘.hic’ (Durand *et al.*, 2016). Second, recent tools designed to handle large mammalian datasets with Kb bin size resolution are mostly based on python, Java or C/C++ programs (Durand *et al.*, 2016; Mendelson Cohen *et al.*, 2017). Although these informatic choices obtain good computing performances, they are not able to easily interact with other resources for biology. In particular, they are not easily incorporated in data analysis workflows based on Bioconductor, i.e. the large collaborative project that over the past 16 years has built a remarkable set of inter-operable tools for the computational genomics community (Gentleman *et al.*, 2004). Third, even the most sophisticated, comprehensive and user-friendly pipelines for Hi-C data analyses (Durand *et al.*, 2016; Serra *et al.*, 2017; Wolff *et al.*, 2018) do not have functionalities allowing basic data manipulations on high-resolution datasets. Basic operations such as ‘access’, ‘subset’ or ‘merge’ are needed to perform additional downstream statistical analyses targeted to data subsets or to build custom pipelines.

**Fig. 1.**
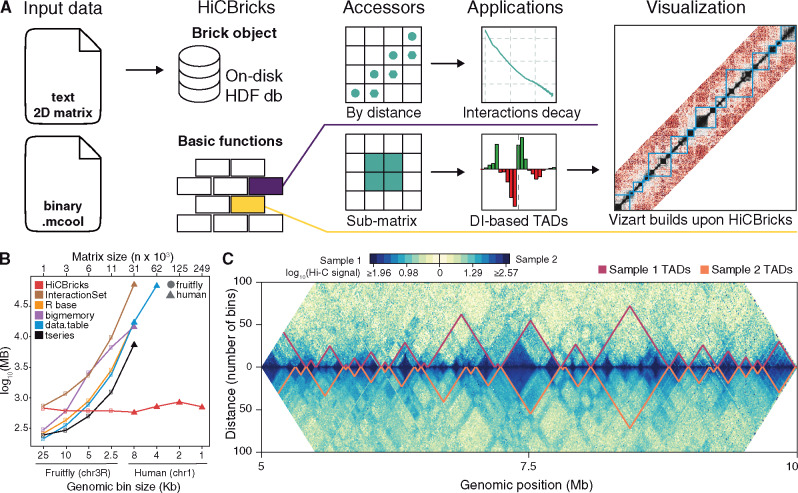
HiCBricks features and performances. (**A**) The cartoon shows HiCBricks framework main features. Input data can be 2D matrices text files or binary file formats (.cool or .mcool). The basic built-in data handling functions of HiCBricks provide the power and flexibility to perform custom analyses. The data accessors to retrieve interactions separated by a given distance allows retrieving diagonals that can be used for example to compute the interaction signal decay with distance. The data accessor functions can be used to build more complex analyses, as in the representative examples highlighting the function to retrieve sub-matrices that is leveraged in two proof of concept functions already included in HiCBricks. Namely functions for calling topological domain borders and sophisticated data visualization. (**B**) Maximum memory usage by HiCBricks and other tools when analyzing contact matrices of increasing size. The maximum memory usage is measured when computing the median interaction signal over 100 diagonals and reported on the y-axis (log scale) in megabytes (MB). The size of the matrices used as input is reported on the top axis as number (*n*) of data rows in an n x n matrix. The matrices are intra-chromosomal contact maps for either a Drosophila chr3R or human chr1. The matrix size corresponds to chromosomes binned at the resolution indicated on the x-axis. For larger matrices some data points are missing for specific tools that could not handle such large data structures in our test. (**C**) A representative data visualization plot that can be obtained with HiCBricks built-in functions. Two representative contact matrices (two samples) are shown in a bipartite (upper versus lower half) 45 degrees rotated heatmap of contact frequencies. A representative set of domain borders is over imposed on each sample

We present HiCBricks, a Bioconductor package providing an efficient, flexible and user-friendly framework for handling large high-resolution Hi-C data matrices. It provides R/Bioconductor users the fundamental bricks for custom Hi-C data analyses. HiCBricks allows importing data in various formats, storing and manipulating them for custom downstream statistical analyses (Fig. 1A). The package is compliant with Bioconductor standards, thus minimizing compatibility issues and maximizing inter-operability with other tools.

## 2 Implementation

HiCBricks accepts contact matrices in multiple formats as input data, including plain text 2D matrices and ‘.mcool’ binary formats. HiCBricks then stores data in on-disk HDF files for efficient data access operations with a number of built-in functions. HiCBricks allows building complete data analysis pipelines, as well as drawing sophisticated plots. See Supplementary Material for additional details on implementation.

## 3 Test cases

HiCBricks is the first Bioconductor package specifically designed for large high-resolution Hi-C contact matrices. Previous Bioconductor packages include also tools for Hi-C data analysis; however, their main purpose and functionalities are different from HiCBricks. For example, HiTC (Servant *et al.*, 2012) implements workflows for data normalization and visualization, but without HiCBricks flexibility for custom operations on data. InteractionSet (Lun *et al.*, 2016) provides instead lower level data accessors functions, but it is not designed for large high-resolution Hi-C matrices. To this concern, as a test case, we examined HiCBricks performance in loading a large contact matrix and computing median interaction signal over 100 diagonals, i.e. a common operation to assess the interaction signal decay with distance. We compared HiCBricks to R base functions and data structures (read.table and dataframe objects, respectively), as well as R packages designed for handling large data structures (bigmemory, data.table and tseries), in addition to InteractionSet. HiCBricks is superior to the other solutions in analyzing large Hi-C matrices in terms of lower memory footprint (Fig. 1B). HiCBricks is actually the only one able to handle human data at a resolution of few Kb bin size, as the other R-based solutions could not handle the largest matrices in our test on a Linux server with 512 Gb RAM.

As additional test cases, using the HiCBricks framework we implemented a topological domain borders calling procedure based on the directionality index (DI) originally proposed by (Dixon *et al.*, 2012), but adopting a local segmentation of DI, and complex data visualization functions (Fig. 1C). These functionalities are showcased in the package vignette. These should be considered as proof of concept applications showing how complex analysis procedures can be easily implemented starting from HiCBricks functions.

## 4 Conclusion

HiCBricks provides a framework useful for bioinformaticians who aim to build a custom analysis procedure for Hi-C data, or need to integrate Hi-C data analysis into pre-existing R/Bioconductor pipelines. On the other hand, biologists with specific questions that can’t be addressed by standard tools will find HiCBricks functionalities useful to perform targeted statistical analyses. HiCBricks enables easy assembling of custom pipelines, going from Hi-C matrices to rich graphical output plots.

## Funding

AIRC Start-up grant 2015 [16841 to F.F.] and AIRC fellowship [21012 to K.P.]

*Conflict of Interest*: none declared.

## Supplementary Material

btz808_Supplementary_Data
