# Supplementary material for: HiCBricks: building blocks for efficient handling of large Hi-C datasets
Source: Bioinformatics. 2019 Nov 7;36(6):1917–9. doi: 10.1093/bioinformatics/btz808 (PMC7703765; doi:10.1093/bioinformatics/btz808)
Supplement: btz808_Supplementary_Data [file btz808_supplementary_data.docx]

**Supplementary information for**

**HiCBricks: building blocks for efficient handling of large Hi-C datasets**

Koustav Pal^1^, Ilario Tagliaferri^1^, Carmen Maria Livi^1^ and Francesco Ferrari^1,2,^*

^1^ IFOM, the FIRC Institute of Molecular Oncology, Milan, Italy

^2^ Institute of Molecular Genetics, National Research Council, Pavia, Italy

* Correspondence: francesco.ferrari@ifom.eu

**Table of contents**

Supplementary Notes 2

Availability 2

Implementation 2

References 4

# Supplementary Notes

## Availability

HiCBricks is implemented as an R/Biconductor package. The most updated stable version is available at: http://bioconductor.org/packages/devel/bioc/html/HiCBricks.html

The package includes also a vignette with detailed tutorial for end-users, that can also be browsed on-line:

https://bioconductor.org/packages/devel/bioc/vignettes/HiCBricks/inst/doc/IntroductionToHiCBricks.html

## Implementation

Input data.

HiCBricks accepts as input Hi-C contact matrices in multiple formats, including plain text 2D matrices and cooler binary formats (.cool or .mcool file extensions).

The loading of 2D text matrices is implemented as a fast and efficient function even for large datasets. However, when loading high-resolution contact matrices from text files, end-users may face highly sparse matrices. As the contact frequency diminishes with distance, the Hi-C signal is expected to become very sparse after a certain diagonal. For this reason, HiCBricks also allows end-users to choose between loading the complete 2D matrices or loading only up to a certain distance from the diagonal.

The cooler binary format (.cool files) is used, among others, also by the 4DNucleome consortium data portal to distribute Hi-C results, thus allowing their easy usability in HiCBricks. If multiple data resolutions or normalization factors are saved in cooler format (.mcool files), then users are able to select their resolution and normalization of choice when loading data into HiCBricks, as well as to load multiple resolutions. We must note that over time the cooler library has changed the format of its output binary files. However, HiCBricks functions for loading cooler data files are able to properly take into account the differences between alternative data file formats as well as the differences between .cool and .mcool files.

The package vignette contains more details on the input data format along with a detailed tutorial with examples about how to load data in HiCBricks. The vignette also shows an example of how to use data in .hic binary format (Durand, et al., 2016).

Data storage and representation

HiCBricks leverages HDF (Hierarchical Data Format) files to allow efficient handling of large Hi-C contact matrices. HiCBricks implements a Hi-C specific HDF data structure, referred to as a *Brick object* and presents accessor functions allowing users to access and manipulate the data.

The *Brick object* implement a HDF structure containing 3 types of information including the Hi-C contact matrices, the reference bin table and optional user-defined genomic annotations:

1. The Hi-C **contact matrix** is defined as a complete 2D matrix for each chromosome or chromosome-pair. The contact matrix for the *cis* (intra-chromosomal) contacts for each chromosome is square n x n dimensional matrix. On the other hand, the *trans* (inter-chromosomal) contacts for each chromosome pair is a rectangular n x m dimensional matrix. The contact matrix loaded into a *brick object* can include the whole genome, or only specfic chromosomes selected by the end-user.
2. The **bin table** is defined as the set of genomic intervals (chromosome, start, end) associated to each row or column of the contact matrix. Hi-C data are usually aggregated and summarized over relatively large genomic intervals (bins) to achieve a more robust quantification of signal (read counts or normalized read counts). These can be defined as either fixed size bins or variable size bins. The latter may be useful for example to handle very high-resolution Hi-C contacts at single restriction fragment resolution.
3. **Annotations** (optional) can be specified by the end-user and associated to the Hi-C data. This information may be, but is not limited to, Topologically Associated Domains (TAD) calls, peak calls for other chromatin marks or gene expression data. In principle, if the annotation information can be represented as a set of genomic intervals it can be stored in the *Brick object*.

HiCBricks associates contact matrices to a reference bin table to facilitate some operations on data and to solve technical problems related to data portability and interoperability. For example, the bin table allows user-friendly functionalities, such as fetching sub-matrices using a query with human readable patterns, e.g. in the form of "chr1:1:1000000".

Then, when importing Hi-C matrices preprocessed by different analysis pipelines, the explicit definition of a reference bin table allows effective exceptions handling. For example, chromosome ends are considered differently in distinct Hi-C analysis pipelines: some assign the chromosome end to fixed-size bins by using a ceiling approximation whereas others use rounding of the actual chromosomal coordinates. Unknowingly, users may not account for these differences, yet they may have unpredictable effects on downstream analyses if not handled properly. By keeping track of the bin table, matrices are always forced to have a consistent dimension.

Another technical detail, often overlooked but with potential bias producing effect, is the way different Hi-C pipelines annotate and handle genomic bins intervals. For example, if the “end” coordinate of each genomic bin corresponds to the “start” coordinate of the subsequent neighbouring bin, then there may be a 1 bp overlap in the genomic intervals associated to adjacent bins. The default behaviour of genomic ranges (GRanges) objects in R (Lawrence, et al., 2013) is to consider overlaps of any size when comparing genomic intervals and it would find an overalp between adjacent bin pairs. HiCBricks, implicitly enforces the use of a bin table with a 1 bp shift in the start position to overcome this technical issue that may generate different results depending on the formatting details of input data. When the end-user performs a data fetch operation with human readable pattern queries, as defined above, HiCBricks correctly handles the exceptions due to 1 bp shift in the start position. These constraints meant to facilitate the adoption of best practices by end-users can also be turned off by more experienced users if needed.

Basic operations on data

HiCBricks offers a number of functions to perform operations on data. The main ones are designed for fetching subsets of data defined as interactions between bin pairs separated by a specific distance, a matrix subset, a specific row or column in the contact matrix, or further subsets of those rows or columns. These operations are implemented as fast and user-friendly functions which allows performing complex data manipulations with a limited memory footprint also on very large data matrices (Figure 1B).

Building more complex data analysis operations with HiCBricks.

The core of HiCBricks storage and retrieval functions is meant to provide the framework to build sophisticated data analysis solutions in a modular way. As an example application, we implemented a custom Topologically Associating Domains (TAD) calling algorithm which is a modification of the method originally proposed by (Dixon, et al., 2012) (see HiCBricks package vignette for details).

HiCBricks also allows building elegant data visualization functions. As an example, we implemented methods for plotting with a single R command line i) a squared heatmap of contact frequencies for one sample; ii) a bipartite two samples heatmap (with a sample in the upper and one in the lower triangle); iii) a 45 degrees rotated contact matrix, i.e. the triangular layout of the contact matrix, in either the one sample or the bipartite two samples version (Figure 1C); iv) as well as all of these options with additional custom annotations such as TAD borders. All of these drawing functions can be coupled with user-defined operations on the data, for example changing from linear to log scale data or capping the range of displayed values, while taking advantage of all the plotting features available in R. Thus, with a single line of code the users are able to obtain high quality sophisticated plots. Examples for all of these plotting functions are shown in details in the Bioconductor package vignette tutorial.

# References

Dixon, J.R.*, et al.* (2012) Topological domains in mammalian genomes identified by analysis of chromatin interactions. *Nature*, **485**, 376-380.

Durand, N.C.*, et al.* (2016) Juicer Provides a One-Click System for Analyzing Loop-Resolution Hi-C Experiments. *Cell Syst*, **3**, 95-98.

Lawrence, M.*, et al.* (2013) Software for computing and annotating genomic ranges. *PLoS computational biology*, **9**, e1003118.
